# Disease burden of tuberculosis and post-tuberculosis in Inner Mongolia, China, 2016–2018 — based on the disease burden of post-TB caused by COPD

**DOI:** 10.1186/s12879-023-08375-w

**Published:** 2023-06-14

**Authors:** Caimei Jing, Huiqiu Zheng, Xuemei Wang, Yanling Wang, Yifan Zhao, Sijia Liu, Jing Zhao, Qianqian Du

**Affiliations:** 1 Department of Health Statistics, School of Public Health, Inner Mongolia Medical University, Jinshan Development District, Hohhot, Inner Mongolia 010110 China; 2 Department of Child and Adolescent Health and Health Education, School of Public Health, Inner Mongolia Medical University, Hohhot, Inner Mongolia 010110 China; 3 Center for Data Science in Health and Medicine, School of Public Health, Inner Mongolia Medical University, Jinshan Development District, Hohhot, Inner Mongolia 010110 China

**Keywords:** Tuberculosis, Post-tuberculosis, Burden of disease, Inner Mongolia

## Abstract

**Background:**

Tuberculosis (TB) remains one of the most serious infectious diseases worldwide. China has the second highest TB burden globally, but existing studies have mostly neglected the post-tuberculosis (post-TB) disease burden. This study estimated the disease burden of TB and post-TB in Inner Mongolia, China, from 2016 to 2018.

**Methods:**

Population data were collected from TB Information Management System. Post-TB disease burden was defined as the burden caused by Chronic Obstructive Pulmonary Disease (COPD) occurring after patients with TB were cured. To estimate the incidence rate of TB, standardized mortality rate, life expectancy, and cause eliminated life expectancy, using descriptive epidemiological, abridged life table and cause eliminated life table. On this basis, the Disability-Adjusted Life Years (DALY), Years Lived with Disability (YLD) and Years of Life Lost (YLL) due to TB were further be estimated. The data were analyzed using Excel 2016 and SPSS 26.0. Joinpoint regression models were used to estimate the time and age trends of the disease burden of TB and post-TB.

**Results:**

The TB incidence in 2016, 2017, and 2018 was 41.65, 44.30, and 55.63/100,000, respectively. The standardized mortality in the same period was 0.58, 0.65, and 1.08/100,000, respectively. From 2016 to 2018, the total DALYs of TB and post-TB were 5923.33, 6258.03, and 8194.38 person-years, and the DALYs of post-TB from 2016 to 2018 were 1555.89, 1663.33, and 2042.43 person-years. Joinpoint regression showed that the DALYs rate increased yearly from 2016 to 2018, and the rate of males was higher than that of females. TB and post-TB DALYs rates showed a rising tendency with increasing age (AAPC values were 149.6% and 157.0%, respectively,* P* < 0.05), which was higher in the working-age population and elderly.

**Conclusion:**

The disease burden of TB and post-TB was heavy and increased year by year in Inner Mongolia from 2016 to 2018. Compared with the youngster and females, working-age population and the elderly and males had a higher disease burden. Policymakers should be paid more attention to the patients’ sustained lung injury after TB cured. There is a pressing need to identify more effective measures for reducing the burden of TB and post-TB of people, to improve their health and well-being.

**Supplementary Information:**

The online version contains supplementary material available at 10.1186/s12879-023-08375-w.

## Background

Tuberculosis (TB) is a relatively severe infectious disease caused by *M. tuberculosis*. In 2022, approximately 10.60 million TB-related cases and 1.4 million TB deaths were reported globally, with the mortality of TB being 15%. In China, there were 780,000 new cases of TB, and the number of multiple-drug-resistant TB was 33,000 in 2021 [1]. Although the incidence of TB in China has shown a decreasing trend in recent years, the number of incidences and deaths of TB ranked 2nd among infectious diseases in China in 2021 [2]. What’s more, with rapidly ageing populations in China, the high incidence of TB in the elderly have impacts on all aspects of economies and societies [3]. In addition, a part of the working-age population died due to TB [4]. As the prevalence of infectious diseases increases globally, the World Bank has proposed the use of Disability-Adjusted Life Years (DALY) to assess the severity of the burden of disease, which measures the total number of healthy years lost from illness onset to death. It includes two parts: Years of Life Lost (YLL) due to early death and Years Lived with Disability (YLD) due to disease. Considering the loss of life at the onset of a disease and the loss of life at the time of death, the actual impact of a disease on population health can be comprehensively evaluated. In 2019, about 122 million DALYs were attributable to TB globally [5]. Among the 30 countries with a high disease burden of TB, China ranked 2nd after India [1].

After treatment, people with TB had a high risk of COPD [6]. Studies showed that the risk of COPD was three times higher in patients over 40 years with TB than that in those without TB [7]. Most patients with TB had poor nutritional status and medication compliance and were prone to complications such as COPD after TB infection [8]. About half of the patients with TB developed clinical symptoms of post-tuberculosis (post-TB) lung disease [9, 10]. In 2019, the first internal post tuberculosis symposium presented post-TB lung disease was a heterogenous condition. Post-tuberculosis lung disease is evidence of chronic respiratory abnormality, with or without symptoms, attributable at least in part to previous tuberculosis [11]. A study on the global lifetime burden of disease due to TB showed that the disease burden of TB should include post-TB sequelae, and 58 million DALYs were attributed to post-TB, with about one-third of these DALYs occurring 15 years or more after the first occurrence of TB. In 2019, China had 2.86 million DALYs attributable to post-TB, ranking 6th among the 30 countries with a high TB burden, just behind countries such as India, the Philippines, and South Africa [5]. The result might be related to the fact that previous studies focused only on TB treatment outcomes and ignored the long-term health problems of patients with TB after cure [12]. Over 60% of patients with TB in China live in rural areas. However, rural healthcare workers always lack professional TB diagnosis and treatment capabilities, and patients with TB had lower awareness of prevention and treatment compliance. Therefore, patients were more prone to irregular treatment and even medication interruption, leading to TB recurrence and post-TB lung injury [13, 14].

Inner Mongolia, is an underdeveloped region located in northern China, is economically backward and lacks medical resources. The incidence of TB decreased from 67.5/100,000 to 35.4/100,000 during 2011–2020 [15, 16]. However, there were still 13,000 new cases of TB reported each year. In 2020, the TB incidence in 6 of 12 Inner Mongolia leagues/cities was over 55/100,000. There were more than 100,000 cases of TB in Inner Mongolia from 2011 to 2020. The burden of TB was heavy in Inner Mongolia, but most of the existing studies focused on the prevalence characteristics of TB, such as incidence. There was a lack of studies related to TB and post-TB disease burden are important but neglected. This study aimed to estimate TB and post-TB disease burden by DALYs and to provide a basis for further prevention and control of TB in Inner Mongolia.

## Methods

### Data source

The TB case data were obtained from the TB Information Management System from 2016 to 2018, which collected information on patients with TB in 101 banners/counties in 12 leagues/cities in Inner Mongolia. Population data were from the National Bureau of Statistics and the Inner Mongolia Statistical Yearbook.

### Variable definition

#### TB

Patients were diagnosed according to the Diagnosis for Pulmonary Tuberculosis (WS 288–2017) [17].

#### Disease burden of TB

The burden of disease in this study was caused by TB and post-TB. Increasing evidence suggested that lung injury persists in patients despite TB treatment, leading to chronic lung diseases such as COPD. Previous TB can lead to pathological changes in the lungs leading to airflow limitation, and COPD is a lung disease characterized by airflow limitation. Although COPD is not the most common post-tuberculosis disease, it has the most serious consequences once it occurs. In this study, the disease burden of post-TB was estimated based on the risk weights of COPD. Due to the lack of data on patients with TB who died from COPD after cured, post-TB YLD was used to represent post-TB DALYs. This study estimated the disease burden for each year from 2016 to 2018 by calculating YLD, YLL, and DALYs. YLL is disability adjusted life years, which refers to the years of life lost due to premature death. YLD is years lived with disability, which refers to years of life lost due to disability caused by illness. Based on the risk weight of COPD after tuberculosis in studies, this study estimated the post-tuberculosis YLD of tuberculosis patients from 2016 to 2018. Due to the lack of data on COPD deaths after tuberculosis patients were cured, we only used YLD to represent post-tuberculosis DALY. The burden of post-TB disease may be underestimated. The calculation formulas are as follows:

1. DALYs = YLL + YLD
2. YLL = D × L, D was the number of TB deaths by age group and sex, and L is the value of life lost by age group and sex.
3. YLD = I × D*w* × L, where I was the number of TB incidences; D*w* was the disability weight, reflecting the severity of disability due to the disease, and the value ranges from 0 to 1 (the higher value of disability weight indicates the higher degree of disability); L was the duration of TB disease in patients. In this study, the disability weight for TB was 0.333, and the disease duration was 1.1 years; the disability weight value for COPD was 0.036, and the duration of COPD was 4.3 years [5, 18].

#### Reported TB incidence rate

The incidence of TB was calculated based on the number of patients registered in the TB reporting system in Inner Mongolia from 2016 to 2018 and the population in the corresponding period. The calculation formula was as follows:

$$\mathrm{Reported}\;\mathrm{TB}\;\mathrm{incidence}\;\mathrm{rate}=\mathrm{reported}\;\mathrm{TB}\;\mathrm{cases}\;\mathrm{in}\;\mathrm a\;\mathrm{certain}\;\mathrm{period}\;\mathrm{of}\;\mathrm{time}/\mathrm{the}\;\mathrm{average}\;\mathrm{population}\;\mathrm{of}\;\mathrm{the}\;\mathrm{area}\;\mathrm{at}\;\mathrm{that}\;\mathrm{time}\ast100,000$$

#### TB Standardized Mortality Rate

The TB Standardized Mortality Rate for each year was as follow:

$$\mathrm{Standardized}\;\mathrm{Mortality}\;\mathrm{Rate}=\Sigma({\mathrm N}_{\mathrm i}/\mathrm N){\mathrm p}_{\mathrm i}$$

where p_i_ is the crude mortality rate for each age group (i), N_i_/N is the composition ratio of each age group (i) in the standard population. The sixth census data of China in 2010 was used as the standard population.

#### Life expectancy and cause-eliminated life expectancy

The life expectancy of the 0-year-old population from 2016 to 2018 was estimated by preparing an abridged life table. Cause-eliminated life expectancy in Inner Mongolia from 2016 to 2018 was calculated by compiling an abridged life table method to reflect the extent of the impact of TB on the life of the population.

#### Age

The age division was based on the sixth census data released by the National Bureau of Statistics of China in 2010, and the population was divided into 19 age groups, which were 0, 1–4, 5–9, 10–14, 15–19, 20–24, 25–29, 30–34, 35–39, 40–44, 45–49, 50–54, 55–59, 60–64, 65–69, 70–74, 75–79, 80–84, and ≥ 85 years old [19].

### Statistical analysis

The disease burden of TB was estimated by reported incidence, mortality, DALYs, and DALYs rate. Rates were standardized using standard population (Sixth Nationwide Population Census). Data were cleaned and analyzed using Excel 2016 and SPSS 26.0. The annual percent change (APC) and average annual percentage change (AAPC) in TB were estimated during 2016–2018 to descript TB DALYs rate trends using Joinpoint 4.9.0. *P* < 0.05 was considered statistically significant. TB reported incidence spatial characteristics of 101 banners/counties in Inner Mongolia was performed by ArcGIS 10.8.

## Results

### Reported incidence and mortality of TB

#### Age-specific incidence of TB

**Fig. 1:**
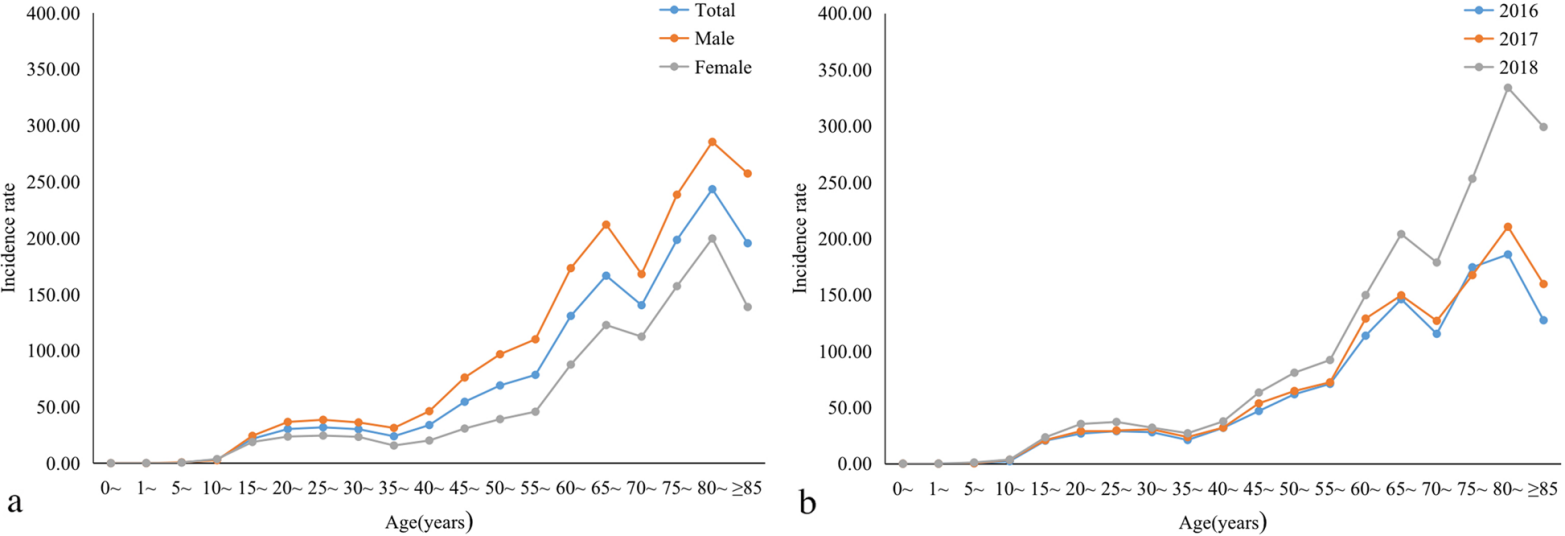
Reported incidence of TB by age in Inner Mongolia, 2016–2018 **a** Age-specific reported incidence by sex **b** Age-specific reported incidence by year. The TB case data were obtained from the TB Information Management System. TB reported incidence was at a low level from 0 to 10 years old, increasing gradually with age after 15 years old. The reported incidence of TB was higher in males than females (Fig. 1a). The highest reported incidence of tuberculosis in 2018

TB reported incidence was at a low level from 0 to 10 years old, increasing gradually with age after 15 years old, rapidly rising after 35 years old, with the first peak at 65–69 years old and the highest peak in the 80–84 years group, with a slight decrease in incidence in the 85 years old and above group. The reported incidence of TB was higher in males than females (Fig. 1a). In 2016–2018, the reported incidence of TB in Inner Mongolia was 47.21/100,000, increased year by year in 2016, 2017, and 2018, which was 41.65, 44.30, and 55.63/100,000, respectively (Fig. 1b).

#### Distribution of TB reported incidence

**Fig. 2:**
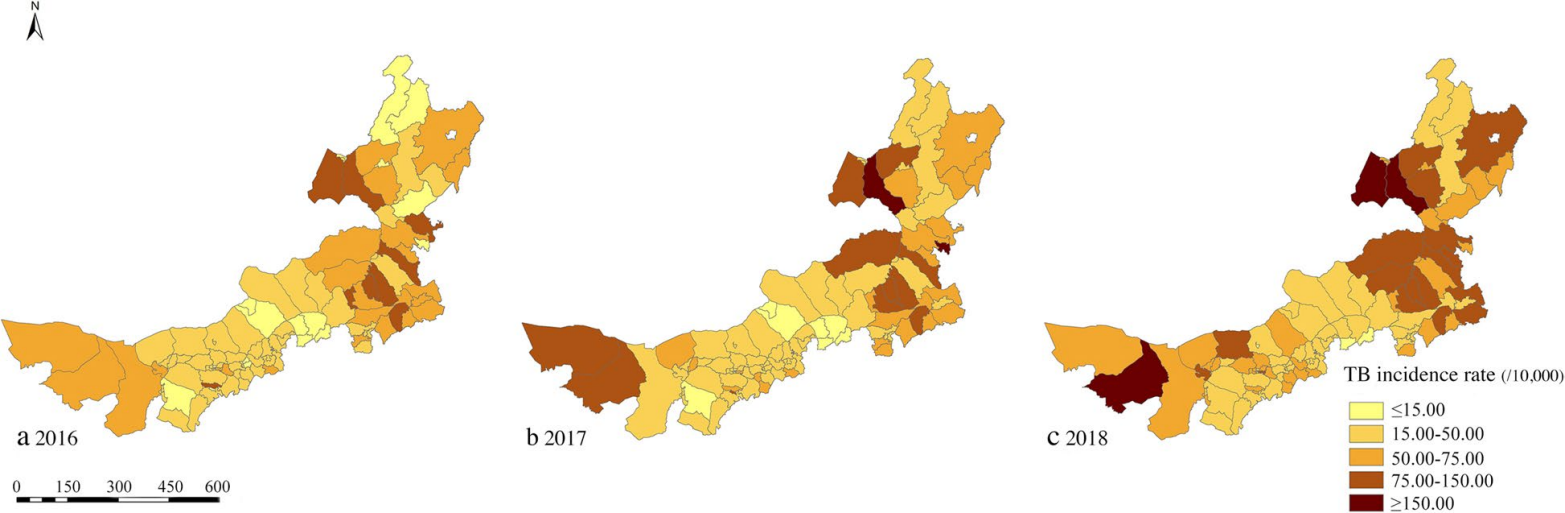
Spatial distribution of TB incidence in Inner Mongolia, 2016–2018 **a** Reported incidence of TB in 2016 **b** Reported incidence of TB in 2017 **c** Reported incidence of TB in 2018. (The digital maps were obtained from the Resource and Environmental Science and Data Center, https://www.resdc.cn/). The TB case data were obtained from the TB Information Management System. Darkest color indicates highest reported incidence rate. The reported incidences in eastern Inner Mongolia were higher than those in the central and western regions

The reported incidences in eastern Inner Mongolia were higher than those in the central and western regions. Among the 101 banners/counties in Inner Mongolia, the highest reported incidence in 2016 was 137.21/100,000 in Naiman Banner (Fig. 2a). In 2017, Xin Barag Zuoqi had the highest reported incidence of 178.57/100,000 (Fig. 2b). In 2018, Jalaid Banner had the highest reported incidence of 943.44/100,000 (Fig. 2c).

#### Standardized mortality of TB

All-cause mortality and TB cause-of-death mortality showed an increasing trend year by year, and the highest rate in 2018 was 1.08 and 0.17/100,000, respectively (Table 1). In 2016–2018, all-cause mortality and TB cause-of-death mortality were higher for males than females (*P* < 0.001).

**Table 1:** TB standardized mortality in Inner Mongolia, 2016–2018 (1/100,000)

Years	Total		Male		Female	
	All cause of death^a^	TB causes of death	All cause of death	TB causes of death	All cause of death	TB causes of death
2016	0.58	0.09	0.75	0.13	0.33	0.04
2017	0.65	0.10	0.84	0.14	0.38	0.03
2018	1.08	0.17	1.36	0.20	0.59	0.11
Total	0.77	0.12	0.99	0.16	0.44	0.06

^a^ All cause of death refers all-cause standardized mortality in patients with tuberculosis. Total refers the total population, regardless of sex. All-cause mortality and TB cause-of-death mortality showed an increasing trend year by year, and the highest rate in 2018

### Life expectancy and cause-eliminated life expectancy

The life expectancy in 2016, 2017, and 2018 was 78.78, 78.53, and 78.57 years, respectively (Table S1). The life expectancy after the elimination of TB increased 0.53, 0.48, and 0.46 years, respectively, and the life span loss rate was 0.67%, 0.61%, and 0.58%, respectively (Table 2). Life expectancy and life expectancy after the elimination of TB were higher for females than for males in 2016–2018.

**Table 2:** Life expectancy of TB eliminated in Inner Mongolia, 2016–2018

Age	2016			2017			2018		
	Total^a^	Male	Female	Total^a^	Male	Female	Total^a^	Male	Female
0 ~	79.31	76.71	82.34	79.01	76.34	82.07	79.03	76.62	81.79
1 ~	78.61	76.01	81.63	78.28	75.64	81.30	78.25	75.86	81.01
5 ~	74.69	72.10	77.72	74.38	71.74	77.39	74.35	71.96	77.10
10 ~	69.75	67.16	72.76	69.43	66.80	72.44	69.40	67.01	72.16
15 ~	64.80	62.22	67.80	64.49	61.87	67.49	64.46	62.08	67.21
20 ~	59.87	57.32	62.85	59.56	56.96	62.54	59.51	57.15	62.24
25 ~	54.95	52.43	57.90	54.63	52.06	57.58	54.57	52.23	57.28
30 ~	50.09	47.62	52.98	49.75	47.22	52.64	49.67	47.35	52.35
35 ~	45.29	42.88	48.10	44.93	42.47	47.74	44.85	42.59	47.45
40 ~	40.47	38.13	43.19	40.11	37.73	42.85	40.02	37.84	42.53
45 ~	35.78	33.56	38.34	35.40	33.12	38.00	35.29	33.19	37.67
50 ~	31.19	29.12	33.56	30.82	28.70	33.22	30.69	28.74	32.91
55 ~	26.85	25.00	28.95	26.52	24.62	28.63	26.36	24.62	28.29
60 ~	22.58	20.96	24.41	22.22	20.55	24.05	22.08	20.59	23.73
65 ~	18.76	17.39	20.28	18.46	17.12	19.93	18.37	17.19	19.63
70 ~	15.31	14.29	16.47	15.05	14.11	16.10	14.97	14.16	15.85
75 ~	11.81	10.94	12.81	11.51	10.72	12.39	11.40	10.77	12.09
80 ~	9.08	8.34	9.92	8.58	7.90	9.34	8.29	7.75	8.89
≥ 85	7.79	6.98	8.67	7.21	6.48	8.00	6.20	7.42	6.79

^a^ Total refers to life expectancy of TB eliminated for the total population, regardless of sex. This study estimated the life expectancy of TB eliminated in Inner Mongolia by compiling abridged life table. To reflect the extent to which TB affects people's lives. The life expectancy of TB eliminated in 2016, 2017, and 2018 was 79.31, 79.01, and 79.03 years, respectively

### Disease burden of TB and post-TB

#### Disease burden of TB

**Fig. 3:**
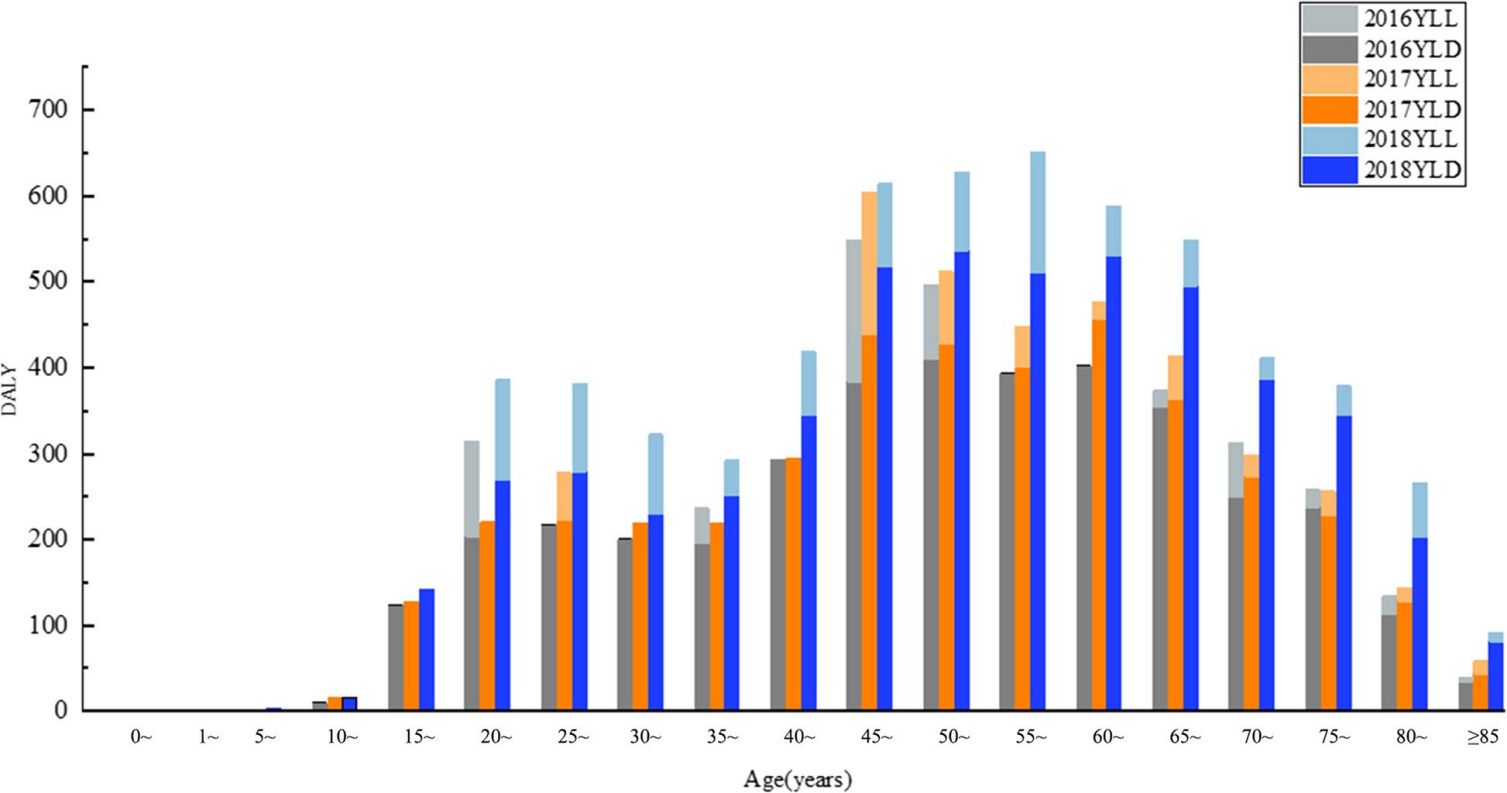
Age-specific disease burden due to TB in Inner Mongolia, 2016–2018. Described using column stacking diagram, the height of each column represents the superimposed value of YLD and YLL, which is DALY. The highest DALYs, YLL, and YLD of TB were in 2018. Abbreviations: YLL, years of life lost; YLD, years lived with disability; DALY, disability-adjusted life years

DALYs of TB from 2016–2018 were 15,114.09 person-years, including 2041.94 person-years of YLL and 13,072.16 person-years of YLD. The YLL of 2016–2018 were 536.68, 505.69, and 999.57, respectively. The YLD of 2016–2018 were 3830.77, 4089.01, and 5152.38, respectively. The highest DALYs, YLL, and YLD of TB were in 2018 (Fig. 3). The disease burden of TB was concentrated in the working-age population. DALYs, YLL, and YLD were higher in males than females in all three years (Table 3).

**Table 3:** Disease burden of TB by gender and age

Age	2016				2017				2018			
	Male DALY	Male DALY rate	Female DALY	Female DALY rate	Male DALY	Male DALY rate	Female DALY	Female DALY rate	Male DALY	Male DALY rate	Female DALY	Female DALY rate
0 ~	0.00	0.00	0.00	0.00	0.00	0.00	0.00	0.00	0.00	0.00	0.00	0.00
1 ~	0.00	0.00	0.37	0.00	0.00	0.00	0.00	0.00	0.00	0.00	0.00	0.00
5 ~	0.73	0.00	0.73	0.00	0.73	0.00	0.73	0.00	2.93	0.00	1.47	0.00
10 ~	3.66	0.01	6.59	0.01	8.42	0.02	7.69	0.02	6.96	0.01	9.89	0.02
15 ~	72.16	0.10	52.75	0.08	76.56	0.11	51.28	0.07	80.59	0.11	62.27	0.09
20 ~	247.74	0.24	68.13	0.07	135.53	0.13	85.35	0.09	217.09	0.21	169.45	0.17
25 ~	139.19	0.13	78.75	0.08	143.22	0.13	135.59	0.13	276.13	0.25	105.86	0.10
30 ~	129.67	0.15	71.79	0.09	146.52	0.16	73.26	0.09	230.72	0.26	92.67	0.12
35 ~	176.37	0.14	60.81	0.05	149.82	0.12	69.96	0.06	216.30	0.18	76.92	0.07
40 ~	208.06	0.17	84.98	0.08	211.72	0.17	83.15	0.07	325.35	0.26	93.77	0.08
45 ~	407.31	0.31	142.13	0.12	454.56	0.34	150.56	0.12	473.98	0.35	141.03	0.11
50 ~	344.26	0.35	153.19	0.17	398.81	0.41	113.55	0.12	424.88	0.43	203.81	0.22
55 ~	266.30	0.30	127.11	0.15	342.40	0.39	105.86	0.12	511.81	0.58	140.66	0.16
60 ~	268.50	0.47	134.80	0.24	332.58	0.58	143.96	0.25	406.52	0.71	182.78	0.32
65 ~	223.81	0.62	150.11	0.38	259.97	0.69	153.77	0.37	323.39	0.82	225.50	0.53
70 ~	204.54	0.60	109.66	0.31	186.88	0.53	112.45	0.30	256.12	0.69	155.31	0.40
75 ~	151.27	0.60	107.59	0.42	174.32	0.67	82.78	0.30	206.96	0.75	172.21	0.61
80 ~	94.54	0.80	39.93	0.34	86.09	0.70	57.65	0.47	135.20	1.05	130.74	1.02
≥ 85	27.46	0.55	12.45	0.22	42.1	0.81	16.85	0.28	56.77	1.04	35.92	0.59

The disease burden of TB was concentrated in the working-age population. DALY and DALY rate of TB were higher in males than females in all three years

*Abbreviations: DALY* Disability-adjusted life years

**Fig. 4:**
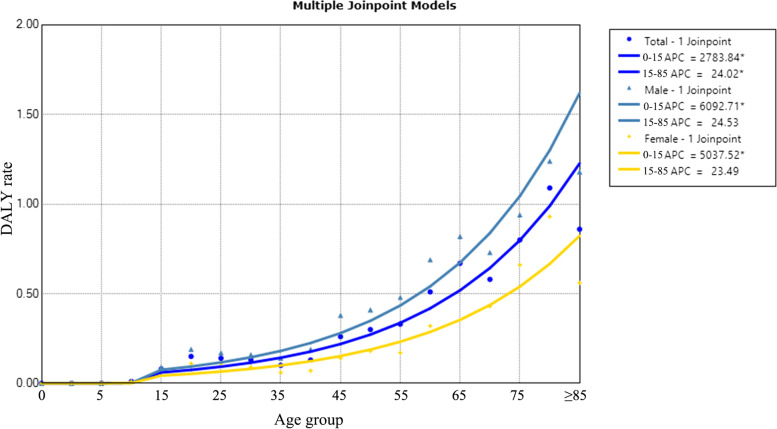
Age-specific trends in TB DALYs rates in Inner Mongolia, 2016–2018. *Indicates *p* < 0.05. Total refers to life expectancy of TB eliminated for the total population, regardless of sex. The solid dot indicates a joinpoint (turning point demarking significance). DALYs rates increased with age. Abbreviations: APC, annual percentage change; DALY, disability-adjusted life years

Age-specific DALYs rates of TB were shown in Fig. 4. DALYs rates increased with age (AAPC_total_ = 149.6%, 95% CI: 84.7–237.2, *P* < 0.001). The DALYs rate increased steeply in males and increased steadily in females, and the DALYs rate was higher for males than for females (AAPC_males_ = 196.7%, 95% CI: 104.3–330.8, *P* < 0.001; AAPC_females_ = 182.8%, 95% CI: 93.7–312.9, *P* < 0.001).

#### Disease burden of post-TB

**Fig. 5:**
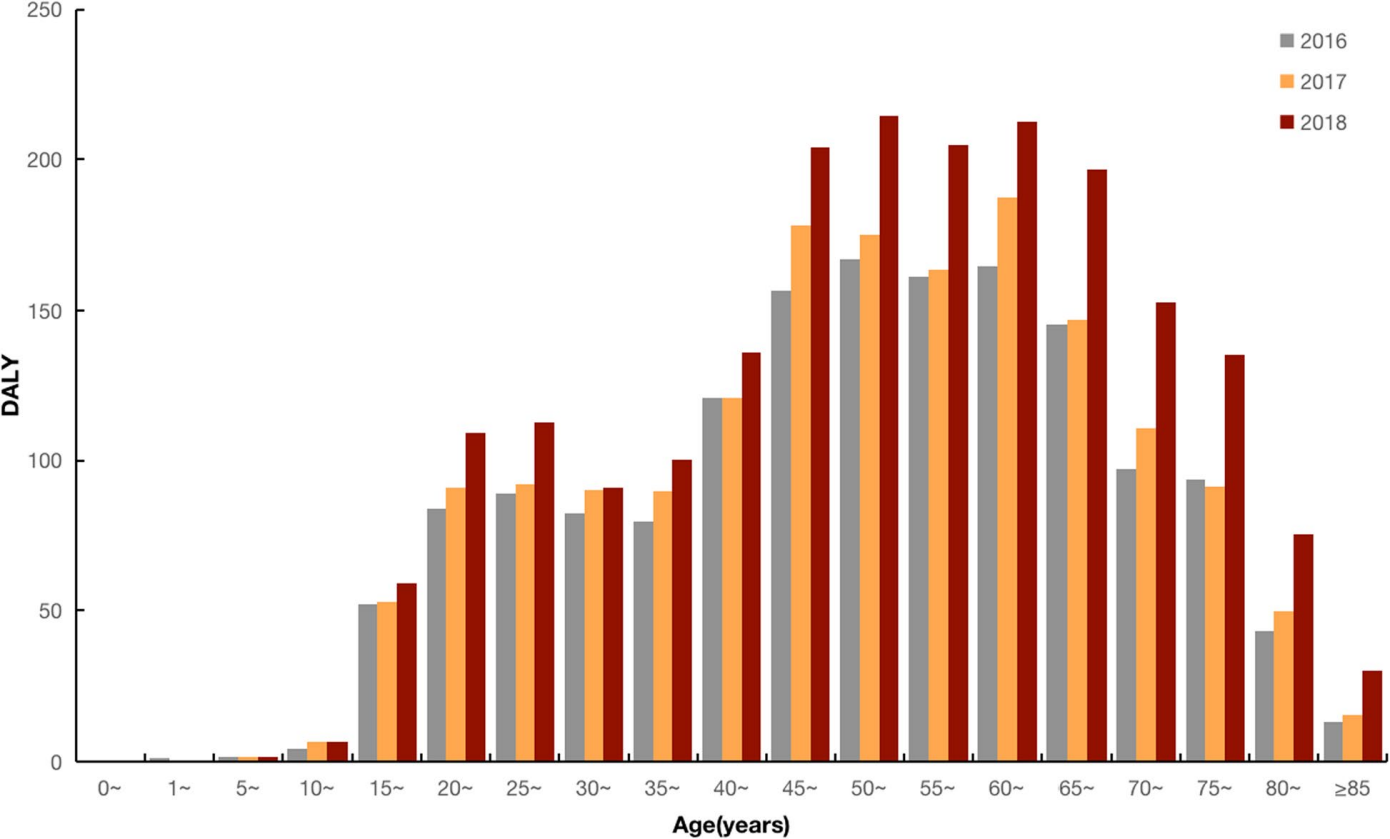
Post-TB disease burden in Inner Mongolia, 2016–2018. The histogram was used to describe, different colors represent different years. The DALYs in males were higher than those in females in three years. Abbreviations: DALY, disability-adjusted life years

The DALYs of post-TB from 2016 to 2018 were 1555.89, 1663.33, and 2042.43 person-years, respectively, with the highest DALYs in 2018. The age-specific post-TB DALYs were mainly concentrated in the 40–79 age group. The DALYs in males were higher than those in females in three years, with males post-TB DALYs being 1.98 times higher than females (Fig. 5 and Table 4).

**Table 4:** Disease burden of post-TB

Age	2016				2017				2018			
	Male DALY	Male DALY rate	Female DALY	Female DALY rate	Male DALY	Male DALY rate	Female DALY	Female DALY rate	Male DALY	Male DALY rate	Female DALY	Female DALY rate
0 ~	0.00	0.00	0.00	0.00	0.00	0.00	0.00	0.00	0.00	0.00	0.00	0.00
1 ~	0.00	0.00	0.15	0.00	0.00	0.00	0.00	0.00	0.00	0.00	0.00	0.00
5 ~	0.31	0.00	0.31	0.00	0.31	0.00	0.31	0.00	1.08	0.00	0.62	0.00
10 ~	1.55	0.00	2.79	0.00	3.41	0.01	3.10	0.01	2.79	0.00	3.72	0.01
15 ~	30.19	0.04	21.98	0.03	32.35	0.04	20.59	0.03	33.44	0.04	25.70	0.03
20 ~	55.88	0.05	28.17	0.03	56.04	0.05	34.83	0.03	65.94	0.06	43.34	0.04
25 ~	56.66	0.05	32.20	0.03	58.98	0.05	32.97	0.03	69.97	0.06	42.88	0.04
30 ~	52.32	0.05	30.19	0.03	59.60	0.06	30.50	0.03	54.49	0.05	36.38	0.04
35 ~	54.64	0.04	25.08	0.02	60.37	0.05	29.57	0.02	69.81	0.05	30.65	0.03
40 ~	85.76	0.06	35.29	0.03	86.84	0.07	34.06	0.03	99.38	0.08	36.53	0.03
45 ~	113.31	0.10	43.19	0.04	131.27	0.11	46.90	0.04	147.99	0.13	55.88	0.05
50 ~	117.34	0.12	49.85	0.06	128.02	0.14	47.21	0.05	158.21	0.17	56.35	0.06
55 ~	108.67	0.14	52.63	0.07	120.43	0.16	43.19	0.06	147.68	0.19	56.97	0.08
60 ~	109.44	0.22	55.42	0.12	128.02	0.26	59.75	0.12	138.55	0.28	73.84	0.15
65 ~	91.18	0.28	54.03	0.16	91.80	0.28	55.26	0.16	121.52	0.37	74.92	0.22
70 ~	59.44	0.20	37.93	0.13	65.17	0.22	45.67	0.16	90.71	0.31	62.07	0.21
75 ~	55.73	0.30	37.77	0.21	58.51	0.31	32.97	0.18	80.50	0.43	54.80	0.30
80 ~	28.02	0.33	15.17	0.19	30.96	0.36	18.73	0.23	41.02	0.48	34.21	0.42
≥ 85	8.51	0.24	4.80	0.12	9.60	0.27	6.04	0.16	19.66	0.55	10.84	0.28

The age-specific post-TB DALYs were mainly concentrated in the 40–79 age group. DALY and DALY rate of post-TB were higher in males than females in all three years

*Abbreviations: DALY* Disability-adjusted life years

**Fig. 6:**
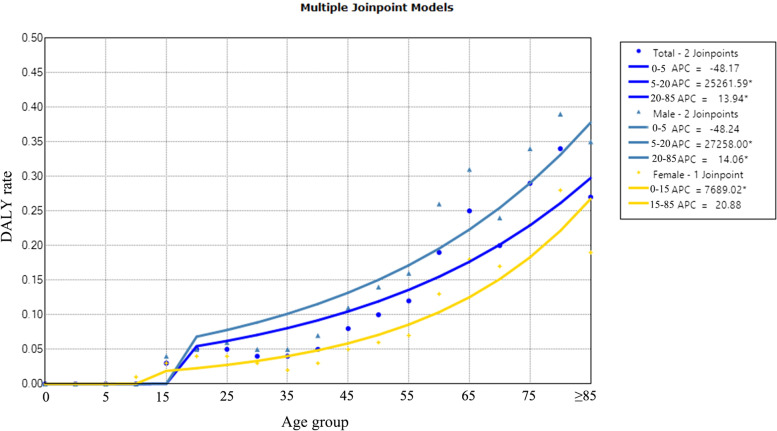
Age-specific trends in post-TB DALYs rates in Inner Mongolia, 2016–2018. *Indicates *p* < 0.05. Total refers to life expectancy of TB eliminated for the total population, regardless of sex. Post-TB DALYs rates increased with age, DALYs rates were lower in males than females before age 20 and increased steeply in males after age 20. The solid dot indicates a joinpoint (turning point demarking significance). Abbreviations: APC, annual percentage change; DALY, disability-adjusted life years

Figure 6 showed the age-specific DALYs rates of post-TB. Post-TB DALYs rates increased with age (AAPC_total_ = 157.0%, 95% CI: 124.6–194.0, *P* < 0.001). DALYs rates were lower in males than females before age 20 and increased steeply in males after age 20 (AAPC_males_ = 160.4%, 95% CI: 128.4–196.9, *P* < 0.001; AAPC_females_ = 205.1%, 95% CI: 94.4–378.6, *P* < 0.001).

#### Total disease burden of TB and post-TB

**Fig. 7:**
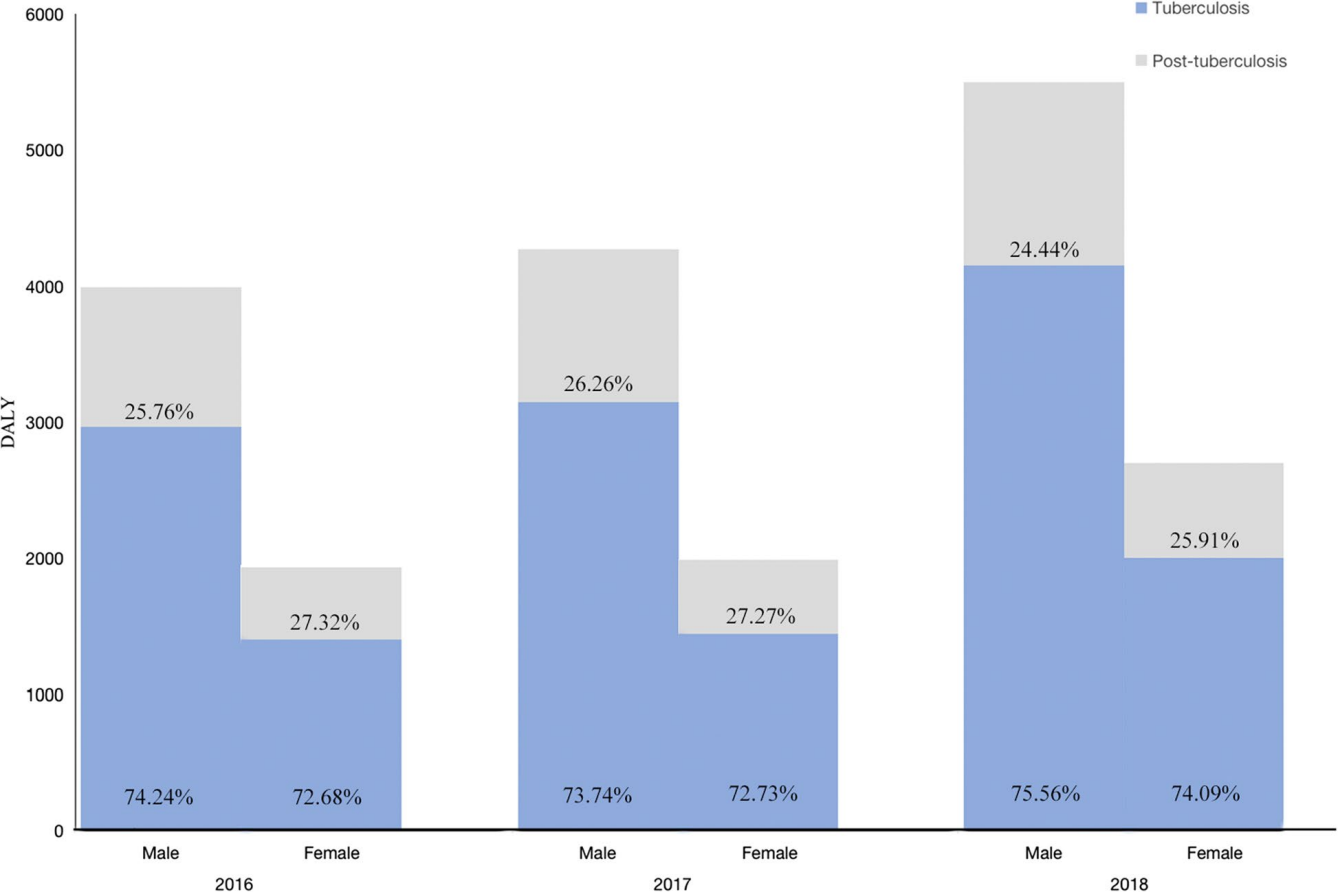
DALYs in TB and post-TB in Inner Mongolia, 2016–2018. Total DALYs with the highest DALYs in 2018. DALYs in post-TB accounted for 25.82% of the total DALYs of TB and post-TB in 2016–2018. Abbreviations: DALY, disability-adjusted life years

Total DALYs of TB and post-TB were 5923.33, 6258.03, and 8194.38 person-years from 2016 to 2018, respectively, with the highest DALYs in 2018. The proportion of DALYs of males was 2.07, 2.15, and 2.03 times that of females in the three years, respectively. DALYs in post-TB accounted for 25.82% of the total DALYs of TB and post-TB in 2016–2018 (Fig. 7).

**Fig. 8:**
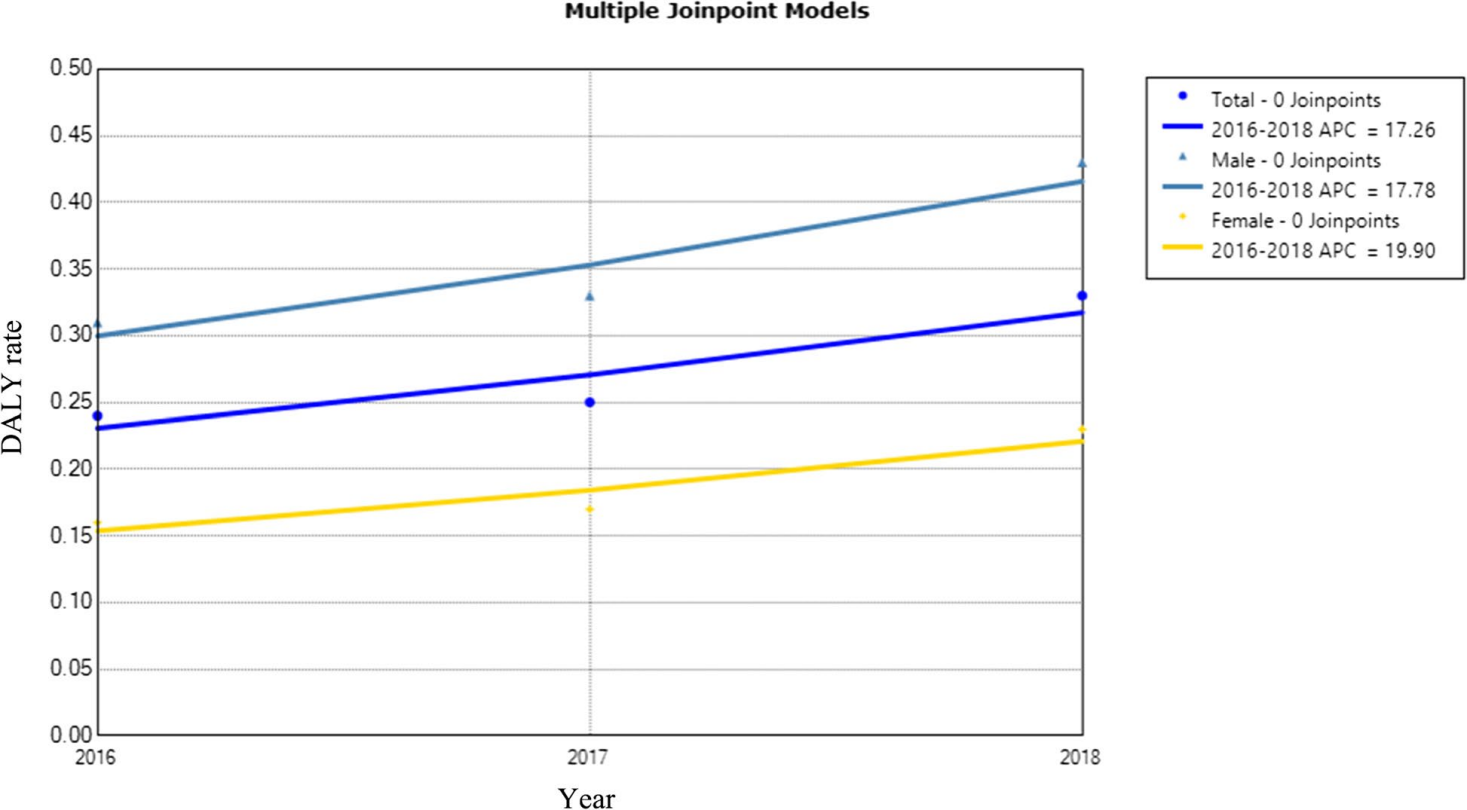
Disease burden of TB and post-TB in Inner Mongolia, 2016–2018. *Indicates *p* < 0.05. Total TB and post-TB disease burden increased with years, DALYs rates of males were consistently higher than that of females. Total refers to life expectancy of TB eliminated for the total population, regardless of sex. The solid dot indicates a joinpoint (turning point demarking significance). Abbreviations: APC, annual percentage change

DALYs rate was 0.24, 0.25, and 0.33/1,000 from 2016–2018, respectively. Total TB and post-TB disease burden increased with years (APC_total_ = 17.3%, 95% CI: -50.8–179.5). The males three-year DALYs rates were 0.31, 0.33, and 0.43/1,000, respectively, and the females were 0.16, 0.17, and 0.23/1,000, respectively (APC_males_ = 17.8%, 95% CI: -43.9–147.2, APC_females_ = 19.9%, 95% CI: -50.6–190.9). DALYs rates of males were consistently higher than that of females (Fig. 8).

## Discussion

China has one of the highest new TB cases globally each year [20]. According to the China National Epidemiological Profile of Statutory Infectious Diseases, TB reported incidence and mortality are the highest among infectious diseases in recent years [2]. In Inner Mongolia, the reported incidence of TB was 47.21/100,000 in 2016–2018, higher than that in Beijing (30.43/100,000) [21]. And the reported incidence rate of tuberculosis in males. Similar to our study, Pereira A et al. showed that the incidence rate of TB was higher in males than in females, and the incidence of TB in males was higher than in the overall rate, and females had the lowest reported incidence rate of TB [22]. We found the standardized mortality was 0.77/100,000 in 2016–2018, higher than that in Zhejiang province (0.19/100,000) [23]. During the same period, the TB incidence in China showed a decreasing trend [24], but our results showed an increasing trend in Inner Mongolia, and the reported incidence of TB was higher in the eastern regions than in the central and western regions. The phenomenon may be related to the lack of knowledge about the transmission and symptoms of TB among the population in Inner Mongolia. The eastern region of Inner Mongolia is agricultural and pastoral area with relatively poor health care resources, where the residents had an insufficient understanding of TB and poor prevention abilities. Furthermore, there was a lack of health care resources. Therefore, it is difficult for people to get treatment in early infection stage [25–27]. In our study, life expectancy after the elimination of TB from 2016 to 2018 increased 0.53, 0.48, and 0.46 years, respectively. Wang et al. reported that the combined loss of life expectancy due to TB in China was 0.11 years [28]. Our study results were higher than those reported by Wang et al., indicating that TB prevention and control in Inner Mongolia was severe.

The total DALYs due to TB in our study consisted of two components: TB and post-TB DALYs. Our study showed that from 2016 to 2018, there were 5923.33, 6258.03, and 8194.38 person-years of DALYs caused by TB and post-TB, with the burden increasing year by year. Moreover, the burden of males was higher than that of females. Poor behavioral habits, such as smoking and drinking more, are prevalent in male patients with TB, and biological gender and high tolerance lead to delayed access to treatment [29–31].

What’s more, we found that YLD of TB accounted for 86.49% of DALYs from 2016 to 2018, and YLD was the major source of the TB disease burden, consistent with Hunan Province and Korea [32, 33]. YLD represents the loss of life due to disease. In our study, YLD meant that patients survived with disability due to TB and lost part of their healthy life. Chinese immunization planning policy requires Bacillus Calmette Guerin (BCG) vaccination during the first 24 h of the neonatal [34]. The duration of protection of the BCG vaccine is usually less than 20 years, and the effectiveness of protection decreased with age [35, 36]. The age-specific analysis in our study showed that the DALYs rate of TB increased from the age of 15 years in this study, which may associate with the immunization program policy. Our results showed that the population above 15 years, the working-age population, and the elderly were the major part of the whole population for TB DALYs. The seventh census of China showed that the population over 65 years old accounted for 13.5% of the total population [37, 38]. The elderly is prone to TB than the younger due to weakened immune system, insufficient nutrient intake, high incidence of chronic diseases, and high prevalence of bad habits (such as smoking and alcohol consumption) [3, 39]. Once infected with TB, the elderly is prone to respiratory complications (e.g., pulmonary infections and COPD) and pathological changes (e.g., lung tissue injury and bronchial dilatation), making TB treatment more difficult [40, 41]. An aging society leads to a decline in the working-age population. When the working-age population infected with TB, the TB symptoms are untypical attributed to strong immune system. Therefore, most young patients with TB fail to receive formal treatment in early infection stage [42]. Previous studies focused on deaths caused by nutrition-deficiency diseases, non-communicable diseases, and injury in the working-age population while neglecting the burden caused by TB [43–45]. However, the working-age population is a major component of social development. When suffering from TB, the work capacities immunity of them would decrease significantly, which would increase the burden on economy and society [46]. Elderly patients with TB always accompanied by low nutrition and poor immunity, leading to delayed lung tissue and airway repair, aggravated lung injury, and increased disease burden due to post-TB [47, 48]. Therefore, it is necessary to focus on the incidence and treatment of TB, and the occurrence of post-TB diseases in TB-curing patients in the working-age and elderly population.

Prior to our study, an Indian study showed that if the impact of post-TB was considered, the disease burden of TB in India would increase by 6.1 million DALYs in 2018 [49]. Menzies NA et.al also pointed out that the disease burden of post-TB accounted for 47% of the total burden, including post-TB [5]. A study pointed out that the global disease burden due to COPD caused by TB was 5.91 million person-years [50], and the global TB disease burden was 45 million person-years during the same period [51], which meant that the disease burden due to COPD caused by TB accounted for 11.8% of the TB disease burden. Although our study only estimated the disease burden of post-TB caused by COPD, the results showed that the disease burden of post-TB accounted for 26.86% of the total disease burden due to TB, higher than the results of the above study. The comparison indicates that the burden of post-TB in Inner Mongolia was heavy. China has increased its efforts to screen for TB [52], but the focus is still on prevention and treatment while neglecting the follow-up of cured patients with TB. Although TB control focused on cutting off the transmission and curing patients, the completion of TB treatment might be the beginning of the chronic respiratory disease [53]. In addition to respiratory symptoms, there was an increased morbidity and mortality of cardiovascular disease in post-patients with TB [54]. These studies suggested that, among those who have been cure of TB, except for COPD, other respiratory diseases, cardiovascular diseases, and neurological damage were also related to the disease burden of post-TB [55]. Therefore, when other systemic diseases caused by post-TB were considered, the burden of post-TB in Inner Mongolia might be significantly heavier than our results. Given the high cost and long treatment period of post-TB, International Union Against Tuberculosis and Lung Disease recommends that patients with TB be evaluated for lung as early as possible and followed up to reduce the possibility of post-TB in patients [56].

Our study was the first to estimate the burden of post-TB in remote China. In recent years, studies on the disease burden associated with TB in China have mostly focused on the economic burden perspective, neglecting the losses due to death, organic disability, and post-TB sequelae in patients with TB, resulting in an underestimation of the disease burden of TB [57–59]. In this study, the disease burden due to post-TB was considered to estimate the health loss of patients caused by TB. However, this study still has some limitations. Firstly, only the disease burden of COPD in post-TB diseases was estimated, and data on mortality of COPD in patients with TB after cure were lacking, leading to possible underestimation of post-TB burden. Furthermore, WHO has reported that about 4 million cases of TB are underreported each year globally, and the number and characteristics of underreported cases in high TB burden countries are still unclear, indicating that underreporting still exists in China. This study did not investigate the underreporting of tuberculosis, which may lead to a certain degree of TB prevalence is undervalued and the burden of TB may be further underestimated [1, 60].

## Conclusion

The disease burden of TB and post-TB was heavy and increased year by year in Inner Mongolia from 2016 to 2018. The disease burden of TB increases gradually with age. Working-age and older populations, especially males, need more attention. Policymakers should pay attention to the burden of post-TB, such as lung injury like COPD, et al. There is a pressing need to establish appropriate assessment measures and standardized follow-up to reduce the risk of TB and post-TB in remote regions with relatively poor medical resources, like Inner Mongolia.

## Supplementary Information

**Additional file 1: Supplementary Table 1.** Life expectancy in Inner Mongolia, 2016-2018.

## Data Availability

The data that support the findings of this study are available from Inner Mongolia Center for Comprehensive Disease Control and Prevention, monitoring data are credible, but restrictions apply to the availability of these data, which were used under license for the current study, and so are not publicly available. Data may however be available from the authors upon reasonable request and with permission of the Inner Mongolia Center for Comprehensive Disease Control and Prevention.

## References

[1] World Health Organization. Global Tuberculosis Report 2022. https://www.who.int/teams/global-tuberculosis-programme/tb-reports/global-tuberculosis-report-2022. Accessed 27 Oct 2022.

[2] Chinese Center for Disease Control and Prevention. 2021 National Statutory Infectious Disease Report. http://www.nhc.gov.cn/jkj/s3578/202204/4fd88a291d914abf8f7a91f6333567e1.shtml. Accessed 22 Apr 2022. (in Chinese).

[3] Yew WW, Yoshiyama T, Leung CC, Chan DP (2018). Epidemiological, clinical and mechanistic perspectives of tuberculosis in older people. Respirology.

[4] Uchimura K, Ngamvithayapong-Yanai J, Kawatsu L, Ohkado A, Yoshiyama T, Ito K, Ishikawa N (2015). Permanent employment or public assistance may increase tuberculosis survival among working-age patients in Japan. Int J Tuberc Lung Dis.

[5] Menzies NA, Quaife M, Allwood BW, Byrne AL, Coussens AK, Harries AD, Marx FM, Meghji J, Pedrazzoli D, Salomon JA (2021). Lifetime burden of disease due to incident tuberculosis: a global reappraisal including post-tuberculosis sequelae. Lancet Glob Health.

[6] Amaral AF, Coton S, Kato B, Tan WC, Studnicka M, Janson C, Gislason T, Mannino D, Bateman ED, Buist S (2015). Tuberculosis associates with both airflow obstruction and low lung function: BOLD results. Eur Respir J.

[7] Shaw JA, Allwood BW (2021). Post-tuberculosis lung disease: exposing the elephant in the room. Afr J Thorac Crit Care Med.

[8] Allwood BW, Byrne A, Meghji J, Rachow A, van der Zalm MM, Schoch OD (2021). Post-tuberculosis lung disease: clinical review of an under-recognised global challenge. Respiration.

[9] Mpagama SG, Msaji KS, Kaswaga O, Zurba LJ, Mbelele PM, Allwood BW, Ngungwa BS, Kisonga RM, Lesosky M, Rylance J (2021). The burden and determinants of post-TB lung disease. Int J Tuberc Lung Dis.

[10] Osman M, Welte A, Dunbar R, Brown R, Hoddinott G, Hesseling AC, Marx FM (2019). Morbidity and mortality up to 5 years post tuberculosis treatment in South Africa: a pilot study. Int J Infect Dis.

[11] Allwood BW, van der Zalm MM, Amaral AFS, Byrne A, Datta S, Egere U, Evans CA, Evans D, Gray DM, Hoddinott G (2020). Post-tuberculosis lung health: perspectives from the First International Symposium. Int J Tuberc Lung Dis.

[12] Ravimohan S, Kornfeld H, Weissman D, Bisson GP (2018). Tuberculosis and lung damage: from epidemiology to pathophysiology. Eur Respir Rev.

[13] Sylvia S, Xue H, Zhou C, Shi Y, Yi H, Zhou H, Rozelle S, Pai M, Das J (2017). Tuberculosis detection and the challenges of integrated care in rural China: a cross-sectional standardized patient study. PLoS Med.

[14] Getnet F, Demissie M, Assefa N, Mengistie B, Worku A (2017). Delay in diagnosis of pulmonary tuberculosis in low-and middle-income settings: systematic review and meta-analysis. BMC Pulm Med.

[15] Implementation Plan of the Action Plan to Contain Tuberculosis in Inner Mongolia (2020–2022). http://wjw.nmg.gov.cn/zfxxgk/fdzzgknr/gfxwj/202105/t20210514_1498120.html. Accessed 22 Jul 2020. (in Chinese).

[16] Shengli L, Lijuan X, Cuixiu W (2021). Eidemiological characteristics of pulmonary tuberculosis patients in Inner Mongolia, 2011–2020. Dis Surveill.

[17] China NHaFPCo (2018). Diagnosis for pulmonary tuberculosis (WS288—2017).

[18] Lange P, Marott JL, Vestbo J, Olsen KR, Ingebrigtsen TS, Dahl M, Nordestgaard BG (2012). Prediction of the clinical course of chronic obstructive pulmonary disease, using the new GOLD classification: a study of the general population. Am J Respir Crit Care Med.

[19] Wang YQ, Li HZ, Gong WW, Chen YY, Zhu C, Wang L, Zhong JM, Du LB (2021). Cancer incidence and mortality in Zhejiang Province, Southeast China, 2016: a population-based study. Chin Med J (Engl).

[20] Glaziou P, Floyd K, Raviglione MC (2018). Global epidemiology of tuberculosis. Semin Respir Crit Care Med.

[21] Yin JF, Huang RW, Jiang H, Gao ZD, Xu WL, He XX (2021). Spatio-temporal distribution of pulmonary tuberculosis and influencing factors in Beijing, 2008–2018. Zhonghua Liu Xing Bing Xue Za Zhi.

[22] Pereira A, Hillesheim D, Silva FMD, Valim RCS, Hallal ALC (2022). Tuberculosis incidence rate time series in the state of Santa Catarina, Brazil: analysis of a decade, 2010–2019. Epidemiol Serv Saude.

[23] Qian W, Yu Z, Kui L, Wei W, Bin C, Songhua C (2022). Epidemiological characteristics of pulmonary tuberculosis in Zhejiang Province from 2016 to 2020. Prev Med.

[24] Jiang H, Liu M, Zhang Y, Yin J, Li Z, Zhu C, Li Q, Luo X, Ji T, Zhang J (2021). Changes in incidence and epidemiological characteristics of pulmonary tuberculosis in Mainland China, 2005–2016. JAMA Netw Open.

[25] Wang Q, Guo L, Wang J, Zhang L, Zhu W, Yuan Y, Li J (2019). Spatial distribution of tuberculosis and its socioeconomic influencing factors in mainland China 2013–2016. Trop Med Int Health.

[26] Lima S, Dos Santos AD, Duque AM, de Oliveira Goes MA, da Silva Peixoto MV, da Conceição AD, Ribeiro CJN, Santos MB, de Araújo K, Nunes MAP (2019). Spatial and temporal analysis of tuberculosis in an area of social inequality in Northeast Brazil. BMC Public Health.

[27] Ma E, Ren L, Wang W, Takahashi H, Wagatsuma Y, Ren Y, Gao F, Gao F, Wang W, Bi L (2015). Demographic and socioeconomic disparity in knowledge about tuberculosis in Inner Mongolia. China J Epidemiol.

[28] Jiansheng W, Xiaoxin H, Shuigao J. Burden of diseases contributed to pulmonary tuberculosis. J Chin Antituberculosis Assoc. 2007;29(03):219–21. (in Chinese).

[29] Thomas BE, Thiruvengadam K, S R, Kadam D, Ovung S, Sivakumar S, Bala Yogendra Shivakumar SV, Paradkar M, Gupte N, Suryavanshi N et al: Smoking, alcohol use disorder and tuberculosis treatment outcomes: a dual co-morbidity burden that cannot be ignored. PLoS One. 2019;14(7):e0220507. 10.1371/journal.pone.0220507 PMC6668833 31365583

[30] Neyrolles O, Quintana-Murci L (2009). Sexual inequality in tuberculosis. PLoS Med.

[31] Chu Y, Soodeen-Lalloo AK, Huang J, Yang G, Chen F, Yin H, Sha W, Huang X, Shi J, Feng Y (2019). Sex disparity in severity of lung lesions in newly identified tuberculosis is age-associated. Front Med (Lausanne).

[32] LiQiong B. Economic burden and impacts of pulmonary tuberculosis patients in Hunan Province. D. Central South University; 2009. (in Chinese).

[33] Jang SY, Kim MJ, Cheong HK, Oh IH (2020). Estimating disability-adjusted life years due to tuberculosis in Korea through to the year 2040. Int J Environ Res Public Health.

[34] Notice of the National Health Commission on the issuance of the National Immunization Program Vaccine Immunization Procedures and Instructions for Children. http://www.nhc.gov.cn/jkj/s3581/202103/590a8c7915054aa682a8d2ae8199e222.shtml. Accessed 12 Mar 2021. (in Chinese).

[35] Nguipdop-Djomo P, Heldal E, Rodrigues LC, Abubakar I, Mangtani P (2016). Duration of BCG protection against tuberculosis and change in effectiveness with time since vaccination in Norway: a retrospective population-based cohort study. Lancet Infect Dis.

[36] Hu Y, Chen Y, Liang H, Wang Y (2018). An overview of coverage of BCG vaccination and its determinants based on data from the coverage survey in Zhejiang Province. Int J Environ Res Public Health.

[37] National Bureau of Statistics of China Bulletin of the Seventh National Census (No.5). http://www.stats.gov.cn/sj/tjgb/rkpcgb/qgrkpcgb/202302/t20230206_1902005.html. Accessed 11 May 2021. (in Chinese).

[38] Zhang CY, Zhao F, Xia YY, Yu YL, Shen X, Lu W, Wang XM, Xing J, Ye JJ, Li JW (2019). Prevalence and risk factors of active pulmonary tuberculosis among elderly people in China: a population based cross-sectional study. Infect Dis Poverty.

[39] Olmo-Fontánez AM, Turner J (2022). Tuberculosis in an aging world. Pathogens.

[40] Xing Z, Sun T, Janssens JP, Chai D, Liu W, Tong Y (2023). Airflow obstruction and small airway dysfunction following pulmonary tuberculosis: a cross-sectional survey. Thorax.

[41] Park HJ, Byun MK, Kim HJ, Ahn CM, Kim DK, Kim YI, Oh JY, Yoon HK, Yoo KH, Jung KS (2018). History of pulmonary tuberculosis affects the severity and clinical outcomes of COPD. Respirology.

[42] Ying X, Jun T, Rui-jun Q, Feng S, Yan-hong Y (2021). Investigation of drug resistance status and risk factors of multidrug-resistance in 249 aged pulmonary tuberculosis patients. Chin J Antituberculosis.

[43] Png ME, Yoong J, Phan TP, Wee HL (2016). Current and future economic burden of diabetes among working-age adults in Asia: conservative estimates for Singapore from 2010–2050. BMC Public Health.

[44] Otsuka T, Takada H, Nishiyama Y, Kodani E, Saiki Y, Kato K, Kawada T (2016). Dyslipidemia and the risk of developing hypertension in a working-age male population. J Am Heart Assoc.

[45] Kristiansen T, Lossius HM, Rehn M, Kristensen P, Gravseth HM, Røislien J, Søreide K (2014). Epidemiology of trauma: a population-based study of geographical risk factors for injury deaths in the working-age population of Norway. Injury.

[46] Birnbaum HG, Morley M, Greenberg PE, Colice GL (2002). Economic burden of respiratory infections in an employed population. Chest.

[47] Li SJ, Li YF, Song WM, Zhang QY, Liu SQ, Xu TT, An QQ, Liu JY, Li HC (2021). Population aging and trends of pulmonary tuberculosis incidence in the elderly. BMC Infect Dis.

[48] Di Gennaro F, Vittozzi P, Gualano G, Musso M, Mosti S, Mencarini P, Pareo C, Di Caro A, Schininà V, Girardi E (2020). Active pulmonary tuberculosis in elderly patients: a 2016–2019 retrospective analysis from an Italian referral hospital. Antibiotics (Basel).

[49] Quaife M, Houben R, Allwood B, Cohen T, Coussens AK, Harries AD, van Kampen S, Marx FM, Sweeney S, Wallis RS (2020). Post-tuberculosis mortality and morbidity: valuing the hidden epidemic. Lancet Respir Med.

[50] Coates MM, Kintu A, Gupta N, Wroe EB, Adler AJ, Kwan GF, Park PH, Rajbhandari R, Byrne AL, Casey DC (2020). Burden of non-communicable diseases from infectious causes in 2017: a modelling study. Lancet Glob Health.

[51] GBD 2017 DALYs and HALE Collaborators (2018). Global, regional, and national disability-adjusted life-years (DALYs) for 359 diseases and injuries and healthy life expectancy (HALE) for 195 countries and territories, 1990–2017: a systematic analysis for the Global Burden of Disease Study 2017. Lancet.

[52] Gilmour B, Xu Z, Bai L, Alene KA, Clements ACA (2022). Risk factors associated with unsuccessful tuberculosis treatment outcomes in Hunan Province. China Trop Med Int Health.

[53] Tiberi S, Torrico MM, Rahman A, Krutikov M, Visca D, Silva DR, Kunst H, Migliori GB (2019). Managing severe tuberculosis and its sequelae: from intensive care to surgery and rehabilitation. J Bras Pneumol.

[54] Romanowski K, Baumann B, Basham CA, Ahmad Khan F, Fox GJ, Johnston JC (2019). Long-term all-cause mortality in people treated for tuberculosis: a systematic review and meta-analysis. Lancet Infect Dis.

[55] Tomeny EM, Nightingale R, Chinoko B, Nikolaidis GF, Madan JJ, Worrall E, Ngwira LG, Banda NP, Lönnroth K, Evans D (2022). TB morbidity estimates overlook the contribution of post-TB disability: evidence from urban Malawi. BMJ Glob Health.

[56] Migliori GB, Marx FM, Ambrosino N, Zampogna E, Schaaf HS, van der Zalm MM, Allwood B, Byrne AL, Mortimer K, Wallis RS (2021). Clinical standards for the assessment, management and rehabilitation of post-TB lung disease. Int J Tuberc Lung Dis.

[57] Huang Y, Huang J, Su X, Chen L, Guo J, Chen W, Zhang L (2020). Analysis of the economic burden of diagnosis and treatment on patients with tuberculosis in Bao'an district of Shenzhen City, China. PLoS ONE.

[58] Liu Y, Xu CH, Wang XM, Wang ZY, Wang YH, Zhang H, Wang L (2020). Out-of-pocket payments and economic consequences from tuberculosis care in eastern China: income inequality. Infect Dis Poverty.

[59] Lu L, Jiang Q, Hong J, Jin X, Gao Q, Bang H, DeRiemer K, Yang C (2020). Catastrophic costs of tuberculosis care in a population with internal migrants in China. BMC Health Serv Res.

[60] Li T, Chen W, Zhao Y, Wang L, Chen M, Du X, Zhang H (2020). Underreporting of notifiable pulmonary tuberculosis cases to the national tuberculosis information management system - China, 2015. China CDC Wkly.

